# JCp-1-NP: New and compiled LA-ICP-MS elemental compositional data of the nano-powdered reference material

**DOI:** 10.1038/s41597-026-07812-0

**Published:** 2026-08-11

**Authors:** Sebastian Flöter, Frank Förster, James Vincent, Tom Sheldrake, Virgil Pasquier, Sebastian Stumpf, Lorenzo Tavazzani, Alexandra Tsay, Elias Samankassou

**Affiliations:** 1 Department of Earth Sciences, University of Geneva, Genève, Switzerland; 2 University of Lausanne, Lausanne, Switzerland; 3 University of Bern, Bern, Switzerland; 4 ETH Zürich, Zürich, Switzerland

**Keywords:** Palaeoceanography, Element cycles, Marine chemistry, Geochemistry, Marine biology

## Abstract

JCp-1-NP is a commercially distributed pressed nano-powder pellet calcium carbonate reference material. It is widely used as a matrix-matched reference material in Laser Ablation-Inductively Coupled Plasma-Mass Spectrometry (LA-ICP-MS) analyses, yet its composition is not well-documented. In this study, we report the uncertainty of the pellet composition by analysing 20 element-to-Ca ratios measured in three different laboratories. The interlaboratory comparison dataset was outlier-corrected and provides a robust assessment of the composition and long-term stability of this nano-powder pressed pellet. In addition, a review of all publicly available compositional data using this reference material published so far is compiled. The dataset is available online at the Figshare repository.

## Background & Summary

In the palaeoclimatology and biomineralisation community, “JCp-1” (GSJ CRM JCp-1)^1^ is the most used and best characterised reference material (RM)^2^. It is analysed either as powdered original GSJ (Geological Survey of Japan) material for solution-based bulk analysis, or as nanoparticulate pressed powder pellet made from reprocessed original GSJ material. To accomplish direct solid analysis of the GSJ powder and to ensure its analytical reproducibility, pressed nano-powder and binder-free pellets have been developed for LA-ICP-MS analysis (e.g.^3^). The commercially distributed JCp-1-NP (myStandards GmbH) is a nano-particulate pressed calcium carbonate powder pellet of the original JCp-1 RM, originating from the skeleton of the scleractinian coral *Porites* sp. It is assumed that the nano-particulate powder shows a different composition compared with the original material due to contamination with material added during the milling process (e.g.^4,5^). JCp-1-NP is an aragonitic powder designed for the determination of major and trace element mass fractions in biologically formed calcium carbonates and used to assess the analytical procedure and quality of LA-ICP-MS measurements. Although JCp-1(-NP) is currently the most used biogenic carbonate reference material, its multielement composition is not very well documented, underlining the importance of an interlaboratory comparison study.

In recent years, JCp-1-NP has been used as a quality control measure in several studies^6–13^. However, the online geological and environmental reference materials (GeoReM) database (https://georem.earth/;^14^) lists - until the publication of this dataset - only one publication for most elements for JCp-1-NP (GeorREM 11377^4^). Due to the scarcity of published data regarding the trace element mass fractions in JCp-1-NP, there is a need for long-term measurements to assess the stability and homogeneity across different measurement conditions.

We present a compilation of all LA-ICP-MS measurements on a single JCp-1-NP pellet (myStandards GmbH, Kiel, Germany) with the batch number: 20170823-60. The size of this batch was in total 60 pellets. The measurements were conducted over three years at the University of Geneva (Switzerland). This work includes the pellet ablation characteristics under frequently used analytical conditions, which are important to reduce mass load of the plasma, avoid downhole fractionation and prevent contamination of the ablation cell. Further it contains a comparison of the composition of the same pellet acquired at two additional laboratories (ETH Zürich and University of Bern, Switzerland). A dataset of 20 element-to-Ca (E/Ca) ratios, ablated with varying laser parameters (spot and line analysis, different spot sizes, laser fluence, repetition rates) over three years, provide a long-term record of measurement uncertainty - comprising trueness and precision of the measurements^15,16^ - of major and trace elements in the carbonate RM. In addition, we summarised analytical LA-ICP-MS results from existing literature to present a dataset of published JCp-1-NP values against which our results are compared.

In summary, we characterised the chemical composition (Table 1) and the ablation efficiency of JCp-1-NP (Table 3). Regarding the ablation characteristics using two laser settings, the ablation depth was 100 to 240 nm per pulse (Table 3) and with that similar to the average aragonitic crystalline reference ablation of ≈230 nm per pulse at 2 J cm^-2^^17^. Regarding the chemical characterisation, we did not observe any long-term temporal trends in measurement uncertainty for any measured E/Ca ratios across the different analytical sessions. Given the instrumental stability that was routinely examined by tuning to the same conditions, our results suggest that the reference material is stable under the chosen storage conditions (i.e., stored dark and dry in an airtight container filled with a desiccant at room temperature).

**Table 1:** Results of the interlaboratory study of JCp-1-NP.

	Parameters		Li/Ca	B/Ca	Na/Ca	Mg/Ca	Al/Ca	K/Ca	Ti/Ca
**Laboratory 1 (University of Geneva)**	Point	*Sample size (n)*	226	230	185	231	226	227	200
		*Median [μmol/mol]*	9.5	403	19120	4335	1905	515	13
		*SD [μmol/mol]*	0.8	46	1398	254	137	45	1
		*SE [μmol/mol]*	0.1	3	103	17	9	3	0
		*2RSD [%]*	16.7	25.8	15.3	13.4	14.2	18.6	16.1
		*LOQ [μmol/mol]*	1.2	5	4	<1	<1	3	<1
	Line	*Sample size (n)*	31	31	19	31	16	15	16
		*Median [μmol/mol]*	8.7	355	17313	4075	1915	485	14
		*SD [μmol/mol]*	0.6	67	829	408	104	57	1
		*SE [μmol/mol]*	0.1	12	190	73	26	15	<1
		*2RSD [%]*	13.2	37.1	9.5	20.0	10.8	24.7	7.8
		*LOQ [μmol/mol]*	0.4	2	3	<1	<1	1	<1
**Laboratory 2 (ETH Zürich)**	Point	*Sample size (n)*	15	15	13	13	14	15	14
		*Median [μmol/mol]*	8.9	552	18763	4082	1935	568	13
		*SD [μmol/mol]*	0.8	57	647	111	87	38	1
		*SE [μmol/mol]*	0.2	15	180	31	23	10	<1
		*2RSD [%]*	18.6	21.3	6.9	5.4	9.0	13.3	15.0
		*LOQ [μmol/mol]*	5.4	10	35	4	5	17	<1
	Line	*Sample size (n)*	14	15	15	15	15	13	15
		*Median [μmol/mol]*	8.9	463	17530	4248	1788	494	13
		*SD [μmol/mol]*	0.6	29	2173	160	253	76	1
		*SE [μmol/mol]*	0.2	7	561	41	65	21	<1
		*2RSD [%]*	12.7	12.1	23.2	7.5	27.3	28.9	14.7
		*LOQ [μmol/mol]*	1.4	3	50	2	9	2	2
**Laboratory 3 (University of Bern)**	Point	*Sample size (n)*	19	19	19	19	19	19	19
		*Median [μmol/mol]*	8.9	431	17454	3815	2005	334	15
		*SD [μmol/mol]*	0.6	15	502	127	42	54	1
		*SE [μmol/mol]*	0.1	3	115	29	10	12	<1
		*2RSD [%]*	13.5	7.0	5.8	6.6	4.2	32.4	10.1
		*LOQ [μmol/mol]*	<0.1	1	<1	<1	<1	11	<1
**Literature values (Figshare table 6**^19^**)**									
*Number of publications (n)*			2	4	4	8	4	2	2
*Reported range [μmol/mol]*			6.5–8.2	428–492	16843–20000	3315–4723	1406–1900	482–530	10–17

**Table 2:** Results of the interlaboratory study of JCp-1-NP (continued).

	Parameters		V/Ca	Mn/Ca	Co/Ca	Cu/Ca	Zn/Ca	Sr/Ca	Y/Ca
**Laboratory 1 (University of Geneva)**	Point	*Sample size (n)*	89	210	194	223	198	229	226
		*Median [μmol/mol]*	0.54	3.1	0.56	0.9	1.4	8667	0.42
		*SD [μmol/mol]*	0.03	0.2	0.05	0.1	0.2	180	0.03
		*SE [μmol/mol]*	<0.01	<0.1	<0.01	<0.1	<0.1	12	<0.01
		*2RSD [%]*	11.9	14.6	17.4	19.5	27.2	4.0	15.3
		*LOQ [μmol/mol]*	0.01	0.1	0.02	0.1	0.2	<1	<0.01
	Line	*Sample size (n)*	15	30	18	18	17	30	31
		*Median [μmol/mol]*	0.54	2.9	0.55	0.8	1.2	8736	0.42
		*SD [μmol/mol]*	0.02	0.2	0.04	<0.1	0.1	94	0.04
		*SE [μmol/mol]*	<0.01	<0.1	0.01	<0.1	<0.1	17	0.01
		*2RSD [%]*	6.4	12.3	12.9	9.8	19.9	2.1	20.4
		*LOQ [μmol/mol]*	0.01	<0.1	0.01	<0.1	0.1	<1	<0.01
**Laboratory 2 (ETH Zürich)**	Point	*Sample size (n)*	14	14	13	15	13	15	13
		*Median [μmol/mol]*	0.52	3.1	0.61	0.9	1.6	8108	0.40
		*SD [μmol/mol]*	0.02	0.2	0.04	0.1	0.2	177	0.02
		*SE [μmol/mol]*	0.01	<0.1	0.01	<0.1	0.1	46	<0.01
		*2RSD [%]*	9.2	11.1	12.8	11.9	24.3	4.4	8.2
		*LOQ [μmol/mol]*	0.04	0.3	0.31	0.5	0.5	<1	<0.01
	Line	*Sample size (n)*	14	14	2	6	15	13	14
		*Median [μmol/mol]*	0.54	3.0	1.08	1.1	1.8	8278	0.41
		*SD [μmol/mol]*	0.01	0.2	0.05	0.2	0.4	65	0.02
		*SE [μmol/mol]*	<0.01	<0.1	0.03	0.1	0.1	18	0.01
		*2RSD [%]*	4.2	11.3	8.8	37.6	37.9	1.6	9.8
		*LOQ [μmol/mol]*	0.05	0.5	0.87	1.1	0.8	<1	0.01
**Laboratory 3 (University of Bern)**	Point	*Sample size (n)*	19	19	17	18	19	18	17
		*Median [μmol/mol]*	0.56	3.0	1.10	0.9	1.6	8404	0.48
		*SD [μmol/mol]*	0.03	0.1	0.07	0.1	0.2	127	0.02
		*SE [μmol/mol]*	0.01	<0.1	0.02	<0.1	<0.1	30	0.01
		*2RSD [%]*	10.5	6.6	13.0	19.3	18.9	3.0	9.6
		*LOQ [μmol/mol]*	0.01	0.2	0.01	<0.1	0.4	<1	<0.01
**Literature values (Figshare table 6**^19^**)**									
*Number of publications (n)*			3	7	1	5	4	8	4
*Reported range [μmol/mol]*			0.43–0.53	1.6–2.5	0.53	0.8–1.2	1.6–2	7653–8814	0.34–0.41

**Table 3:** Results of the interlaboratory study of JCp-1-NP (continued).

	Parameters		Ba/Ca	La/Ca	Ce/Ca	Pb/Ca	Th/Ca	U/Ca
Laboratory 1 (University of Geneva)	Point	*Sample size (n)*	225	158	160	227	83	232
		*Median [μmol/mol]*	8.8	0.08	0.11	0.16	0.023	1.13
		*SD [μmol/mol]*	0.6	0.01	0.01	0.01	0.002	0.05
		*SE [μmol/mol]*	<0.1	<0.01	<0.01	<0.01	<0.001	<0.01
		*2RSD [%]*	14.1	23.3	25.4	16.7	20.4	9.5
		*LOQ [μmol/mol]*	<0.1	<0.01	<0.01	<0.01	<0.001	<0.01
	Line	*Sample size (n)*	31	3	3	28	3	31
		*Median [μmol/mol]*	8.3	0.07	0.12	0.15	0.022	1.09
		*SD [μmol/mol]*	0.5	<0.01	0.01	0.01	0.002	0.06
		*SE [μmol/mol]*	0.1	<0.01	0.01	<0.01	0.001	0.01
		*2RSD [%]*	12.5	12.1	19.7	11.6	16.1	10.9
		*LOQ [μmol/mol]*	<0.1	<0.01	<0.01	<0.01	<0.001	<0.01
**Laboratory 2 (ETH Zürich)**	Point	*Sample size (n)*	14	15	15	15	15	15
		*Median [μmol/mol]*	8.7	0.07	0.11	0.18	0.023	1.24
		*SD [μmol/mol]*	0.7	0.01	0.02	0.01	0.003	0.05
		*SE [μmol/mol]*	0.2	<0.01	<0.01	<0.01	0.001	0.01
		*2RSD [%]*	16.6	20.2	27.2	10.7	23.2	8.1
		*LOQ [μmol/mol]*	<0.1	<0.01	<0.01	0.01	<0.001	<0.01
	Line	*Sample size (n)*	14	13	15	15	15	12
		*Median [μmol/mol]*	8.7	0.08	0.12	0.17	0.021	1.20
		*SD [μmol/mol]*	0.4	<0.01	0.02	0.01	0.002	0.03
		*SE [μmol/mol]*	0.1	<0.01	<0.01	<0.01	0.001	0.01
		*2RSD [%]*	10.3	10.0	31.6	12.8	19.0	5.8
		*LOQ [μmol/mol]*	<0.1	<0.01	<0.01	<0.01	<0.001	<0.01
**Laboratory 3 (University of Bern)**	Point	*Sample size (n)*	18	18	17	19	19	18
		*Median [μmol/mol]*	9.1	0.09	0.13	0.17	0.028	1.25
		*SD [μmol/mol]*	0.3	0.01	0.01	0.02	0.003	0.04
		*SE [μmol/mol]*	0.1	<0.01	<0.01	<0.01	0.001	0.01
		*2RSD [%]*	6.3	20.8	16.0	19.2	21.5	6.1
		*LOQ [μmol/mol]*	<0.1	<0.01	<0.01	0.07	<0.001	<0.01
**Literature values (Figshare table 6**^19^**)**								
*Number of publications (n)*			6	NA	3	6	4	4
*Reported range [μmol/mol]*			6.4–7.6	NA	0.06–0.09	0.13–0.16	0.010–0.021	0.98–1.11

The precision, expressed as two times the relative standard deviation (2RSD) calculated from the arithmetic mean, was generally <30% (2RSD). The interlaboratory comparison showed no systematic deviation between Laboratories 1, 2 and 3, with most E/Ca displaying a relative bias (RB), calculated as $\frac{|\bar{x}-{x}_{{ref}}|}{{x}_{{ref}}}\ast 100$, of less than 10%. Compared to published data, our E/Ca ratios deviate by ≈23% ± 17% (RB) from those of Jochum *et al*.^4^, by ≈15% ± 30% (RB) from the manufacturer’s specified values, and on average by 12% ± 11% (RB) from other published literature values^6–13^.

This dataset was acquired by using the same commercially well distributed pellet over more than three years with different instrumental settings and in three different laboratories providing insights into the possible deviations arising from the different measurement conditions. The amount of data points together with the diversity of set ups in addition to a strictly uniform evaluation of the results provide a robust dataset for the carbonate reference material JCp-1-NP.

## Methods

### LA-ICP-MS analyses

#### Long-term study in Laboratory 1

Measurements were performed over three years (19.11.2021 to 27.09.2024) with a 193 nm ArF excimer laser ablation system (ESL 193 HE) coupled to an Agilent 8900 triple quadrupole inductively coupled plasma mass spectrometer (LA-ICP-MS) at the University of Geneva (Laboratory 1). During the evaluation period, spot as well as line scans were carried out on the pellet. The instrument was routinely tuned on NIST SRM 610 silicate glass^18^ while using a 40 μm circular spot, an ablation rate of 10 Hz, and a laser fluence of 7 J cm^−2^. A ThO/Th ratio (indicating oxide formation) of <0.3%, a mass 21/mass 42 ratio (doubly charged interferences) of <0.3% and a U^+^/Th^+^ ratio (element fractionation) of 1.00 to 1.05 was achieved in single quadrupole no-gas mode. The laser ablation cell was flushed with 800–850 ml min^−1^ He as carrier gas while Ar was used as the nebuliser gas. For signal smoothing the sampling gas was flowing through a commercial gas mixing device from ESI (Elemental Scientific Lasers).

The mass-to-charge ratios (Figshare Table 1^19^) were selected for the least isobaric, polyatomic and multiple charged ion interferences in LA-ICP-MS analyses of carbonates. The selection was based on mass-to-charge ratio recommendations^20^ for low-mass resolution. A typical static spot analysis consisted of ≈30 s of background acquisition, followed by ≈45 s of signal acquisition and a washout phase using various laser ablation parameters. The dwell-times for the chosen mass-to-charge ratios are reported in Figshare Table 1^19^. The quantification was achieved by using NIST 612 as a primary calibrant^18^. The quantification method was kept the same for all three laboratories.

#### Laboratory 2 and 3

For comparison purposes, the JCp-1-NP (myStandards GmbH, batch number: 20170823-60) was also analysed at ETH Zürich (Laboratory 2) and University of Bern (Laboratory 3) using different instruments and laser settings (Table 4). Laser parameters were kept constant during individual measurements. The dwell-times for the chosen mass-to-charge ratios for both laboratories are reported in Figshare Table 1^19^.

**Table 4:** Overview of the used instruments and their settings for all three laboratories. Value in parentheses shows median.

	Laboratory 1	Laboratory 2	Laboratory 3
**ICP-MS**	Agilent 8900	Thermo Scientific ELEMENT XR	PerkinElmer Elan DRC-e
**Laser**	ESL 193 HE (Elemental Scientific), 193 nm ArF excimer laser	Resonetics RESOlution LR (ASI/Applied Spectra), 193 nm ArF excimer laser	COMPex-Pro (Lambda Physics),193 nm ArF excimer laser
**Laser fluence**	2.0–8.0 (4.0) J cm^−2^	3.5 J cm^−2^	≈10 J cm^−2^
**Ablation chamber**	TwoVol2, two-volume cell (Elemental Scientific)	S-155, two-volume cell (Laurin Technic)	custom-built 20 cm^3^ ablation cell
**Spot size**	ablation line and spot: 10–50 × 100–150 µm and 20–60 (60) µm respectively	ablation line and spot: 43 µm	spot: 90 µm
**Repetition rate**	3–10 (10) Hz	10 Hz	10 Hz
**Line scan speed**	5–20 (10) µm s^−1^	25 µm s^−1^	—
**spots/lines (n)**	88–232/3–31*	15/15	19/0

^*^depending on the E/Ca. Refer to Table 1.

The analyses at ETH Zürich (Switzerland) were performed on 22.11.2024 using a RESOlution LR (ASI/Applied Spectra) 193 nm ArF excimer laser system coupled to an Element XR (Thermo Scientific) sector field ICP-MS. Ablation was executed in a dual-volume, fast-washout S-155 ablation cell (Laurin Technic) and fluxed with a carrier gas (≈0.25 l min^−1^ He) followed by a make-up gas (≈1 l min^−1^ Ar and 2 ml min^−1^ N_2_ to increase the sensitivity). Before introduction into the ICP-MS, the ablated aerosol was homogenised by flushing through an in-house squid device. The instrument was optimised for high sensitivity on Pb, Th and U masses whilst keeping production of oxides (^248^ThO^+^/^232^ThO^+^  ≤0.25%) low and the U/Th ratio at ≈1 (on NIST SRM 612 glass). For the analysis of JCp-1-NP, a spot size of 43 µm with a laser repetition rate of 10 Hz and a laser energy density on the surface of ≈3.5 J cm^−2^ was used. These ablation parameters were applied to static spot and line scan acquisitions (25 µm s^−1^ line scan speed). Before each line scan acquisition, the sample surface was cleansed by a fast scanning (100 µm s^−1^) pre-ablation line (51 µm spot size). Measurements for each line scan consisted of 19 s of background acquisition followed by 40 s of sample ablation. Before each static spot analysis, the sample surface was cleansed by three pre-ablation pulses (51 µm spot size). Measurements for static spot analysis consisted of a 40 s background acquisition followed by 40 s of sample ablation. Per peak 100 samples were recorded with a mass window of 10. A standard bracketing procedure with NIST 612 was applied to quantify the sample results and a synthetic glass GSD-1G used for quality control^21^.

The analysis at the University of Bern (Switzerland) was performed on 17.12.2024 using a GeoLas (Lambda Physik) pulsed 193 nm ArF excimer laser system coupled with a PerkinElmer ELAN DRC-e inductively coupled plasma quadrupole mass spectrometer. Helium was employed as carrier gas at a flow rate of 1.0 l min^−1^, supplemented with 8 ml min^−1^ H_2_ gas before entering the custom-built 18 cm^3^ ablation cell (e.g.^22^). Before introduction into the mass spectrometer, 0.60 l min^−1^ of Ar was added as nebuliser gas to the carrier gas mixture. Following thermal stabilisation of the system, the mass spectrometer was tuned on a NIST SRM 612 glass to maximise signal intensities across the entire mass range while maintaining a ThO/Th ratio below 0.5% and a Th/U sensitivity ratio between 0.97 and 1.00. Tuning was done with a circular laser beam of 64 µm diameter, at a repetition rate of 10 Hz and a fluence of 10 J cm^−2^. The same repetition rate and fluence were used during the analysis of JCp-1-NP. Each spot analysis consisted of a 30-second background measurement, followed by 40 seconds of ablation using a 90 µm beam diameter, and a 40-second washout period. A total of 19 measurements of JCp-1-NP were performed, bracketed by six analyses each of NIST SRM 612 and GSD-1G, with three analyses conducted before and three after the sample sequence for each standard.

### Data processing

Signal processing for all three laboratory datasets was carried out in the same way using RStudio^23^ with R version 4.2.0^24^. ^43^Ca was used as the internal standard element for signal normalisation. Signal and background were selected automatically based on changes in the acquired Ca counts using change point functions^25,26^. Elemental data were normalised to an assumed Ca content of 40.04 wt.% in pure aragonite to avoid any uncertainties resulting from the composition of the powdered material. At the beginning and end of each sequence, the synthetic glass standard NIST SRM 612^18^ was analysed. Mass fractions were calculated as element-to-Ca ratios with NIST SRM 612 for external calibration. Element-specific limit of detections (LOD)^27^ were calculated as:

1 $${LOD}=\frac{3{{\rm{\sigma }}}_{b}}{S}\sqrt{\frac{1}{{n}_{{bg}}}+\frac{1}{{n}_{a}}}$$

With ${{\rm{\sigma }}}_{b}$ the standard deviation of the background counts, S the sensitivity, determined on the calibration material NIST SRM 612, ${n}_{{bg}}$ the background counts, and ${n}_{a}\,$ the analyte counts. If measured mass fractions fell below the calculated LODs, those values were discarded in the data processing. Elemental mass fractions are presented as E/Ca ratios in µmol mol^−1^.

The results of this study were rounded according to the element-specific long-term uncertainty u. The significant digits R were calculated as:

2 $$R=0.7\ast {\log }_{10}u$$

### Outlier detection

Three outlier detection methods were applied to the obtained E/Ca dataset: two parametric approaches assuming normal distribution (Grubbs’ test for larger sample sizes and Dixon’s Q test for smaller sample sizes) and a non-parametric method (modified z-score). Parametric tests are generally robust for larger sample sizes (n > 30–40), even with data not normally distributed^27^. Due to the small sample size for Laboratory 2 (n = 15 for spot and line measurements) and Laboratory 3 (n = 19), we present the results of both parametric and non-parametric tests.

### Parametric tests: Grubbs’ test and Dixon’s Q test

Grubbs’^28^ and Dixon’s^29^ outlier detection tests were performed using PAST (PAleontological STatistics) software v.5.2^30^. The Grubbs’ test statistic is defined as

3 $$G=\frac{(max{\rm{| }}{x}_{i}-\bar{x}{\rm{| }})}{s}$$

where ${x}_{i}$ is the individual sample, $\bar{x}$ is the sample mean, and s the standard deviation. Outliers were identified iteratively, when

4 $$G > \frac{N-1}{\sqrt{N}}\sqrt{\frac{{{t}^{2}}_{\frac{\alpha }{2N}\left(N-2\right)}}{N-2+{{t}^{2}}_{\frac{\alpha }{2N}\left(N-2\right)}}}$$

with N the sample size, and ${{t}^{2}}_{\frac{\alpha }{2N}\left(N-2\right)}$ is the critical value of the t-distribution at a significance level of $\frac{\alpha }{2N}$ with N-2 degrees of freedom. For smaller sample sizes (N ≤ 30), Dixon’s Q test was performed by calculating the test statistic value of the sorted data according to:

5 $${r}_{22{data}min}=\frac{{x}_{3}-{x}_{1}}{{x}_{n-2}-{x}_{1}}$$

6 $${r}_{22{data}max}=\frac{{x}_{n}-{x}_{n-2}}{{x}_{n-2}-{x}_{3}}$$

with x_n_ being the n’th value in the ordered row of datapoints. The value x_1_ is regarded as an outlier if the r_22_ is larger than the critical value for N, the number of observations and the level of significance α (here = 0.05).

### Non-parametric test: Modified z-score

The modified z-score was calculated using R Version 4.2.0^24^ with

7 $$z=0.6745\ast \frac{({x}_{i}-\widetilde{x})}{{MAD}}$$

where $\widetilde{x}$ is the sample median, ${MAD}$ is the median absolute deviation, calculated as ${MAD}={median}(| {x}_{{i}}-\widetilde{x}| )$. Outliers are identified when |z| > 3.5. Trueness was evaluated using the median of samples with outliers removed via the modified z-score, and precision for JCp-1-NP is likewise reported based on the outlier-fitted dataset. Results of the Grubbs’ and Dixon’s Q test are summarised in Figshare Table 5^19^.

### Ablation characteristics

**Fig. 1:**
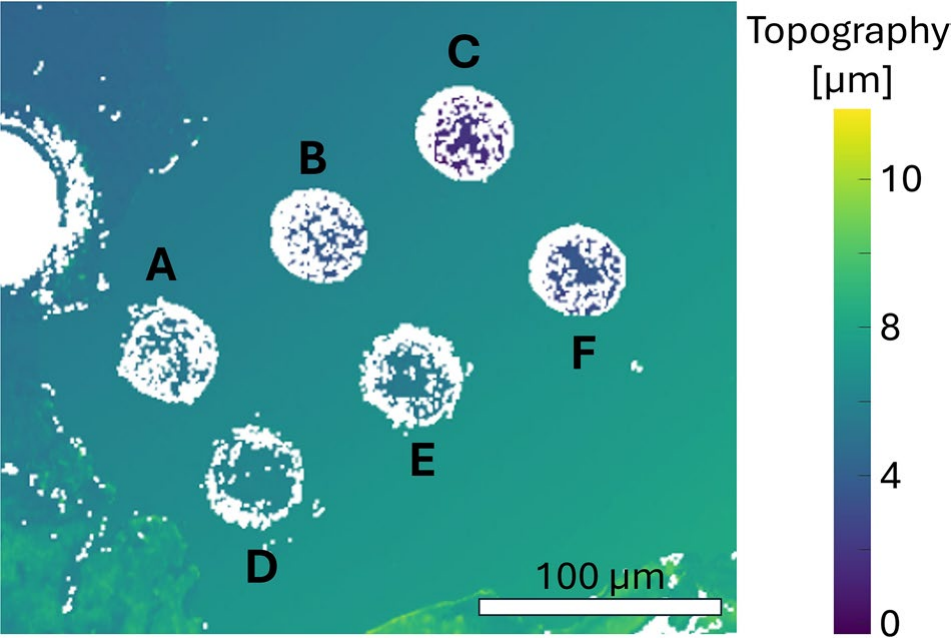
Topography of the pressed nano-powder pellet JCp-1-NP after laser ablation with a set of six different parameter combinations. The white color represents areas where no reliable estimates of the topography could be calculated. A: 5 pulses, 3.5 J cm^−2^ B: 10 pulses, 3.5 J cm^−2^ C: 20 pulses, 3.5 J cm^−2^ D: 5 pulses, 2.5 J cm^−2^ E: 10 pulses, 2.5 J cm^−2^ F: 20 pulses, 2.5 J cm^−2^ (for data see Table 3).

To document the ablation behaviour of the nano-powder pressed pellet a set of six ablation spots was created under typical ablation conditions (Fig. 1). Three different numbers of laser pulses per spot (n = 5, 10 and 20) were combined with two different laser fluences (2.5 and 3.5 J cm^−2^). The surface topography of the ablated area was imaged with a white light interferometer - a Bruker Contour GT – at the University of Lausanne, Switzerland. The software package Vision 64 from Bruker was used to export the surface morphology data. Subsequently, the data were further evaluated with MATLAB to extract an average crater depth. The arithmetical mean of three transects per ablation pit was used to calculate the depth. Each transect was background corrected with the rim values, and about 30 to 40 data points were averaged for each transect for the depth in the pit. The results are summarised in Table 5 and Fig. 1.

**Table 5:** Ablation efficiency of the aragonitic pressed nano-powder pellet JCp-1-NP under different laser ablation settings.

Ablation setting	Laser pulses (n)	Fluence [J cm^−2^]	Crater depth ± SD [µm]	Ablation efficiency ± SD [nm pulse^−1^]
A	5	3.5	0.7 ± 0.2	146 ± 29
B	10	3.5	2.4 ± 0.1	243 ± 10
C	20	3.5	4.6 ± 0.1	229 ± 7
D	5	2.5	0.5 ± 0.1	96 ± 11
E	10	2.5	1.2 ± 0.1	118 ± 2
F	20	2.5	3.5 ± 0.1	173 ± 5

## Data Records

Tables 1–3 show a summary of the raw median concentrations of all three laboratories for line and spot ablation of JCp-1-NP including the numbers of measurement, the analytical precision, LOQ, and the concentration range reported in literature. The LA-ICP-MS setup of all three laboratories is provided in Table 4 and the laser ablation efficiency of laboratory 1 is provided in Table 5. Figshare Table 1^19^ contains the chosen mass-to-charge ratios of all three labs and the corresponding dwell times used. In Figshare Table 2^19^ all raw concentration data points are provided that were used in the data descriptor. While Figshare Table 3^19^ provides the outlier filtered data using the z-score method, Figshare Table 4^19^ shows the Dixons Q filtered data. A summary of the filtered concentration data of all three laboratories is summarised in Figshare table 6^19^. A compilation of all published concentration values of JCp-1-NP is provided in Figshare table 6^19^ while Figshare table 7^19^ contains the relative deviation of the concentrations results of laboratory 1 to published values. The data tables are provided separately as.csv files in addition to a single.xlsx file containing all tables of the data descriptor in the Figshare repository^19^.

## Technical Validation

Measurement uncertainty and internal consistency were assessed on the nano-powder pressed pellet JCp-1-NP (myStandards GmbH, batch number: 20170823-60). Precision (2RSD) was calculated from long-term measurements at Laboratory 1. Trueness is expressed as relative bias (%)^16^, and was determined by comparing the outlier-removed median values to results from the two other labs (Laboratory 2 and Laboratory 3), and to published reference. These reference values were grouped into (i) GeoReM listed values or manufacturer-provided data, and (ii) values reported in studies explicitly using a JCp-1-NP pellet.

### Long-term consistency of measurements

**Fig. 2:**
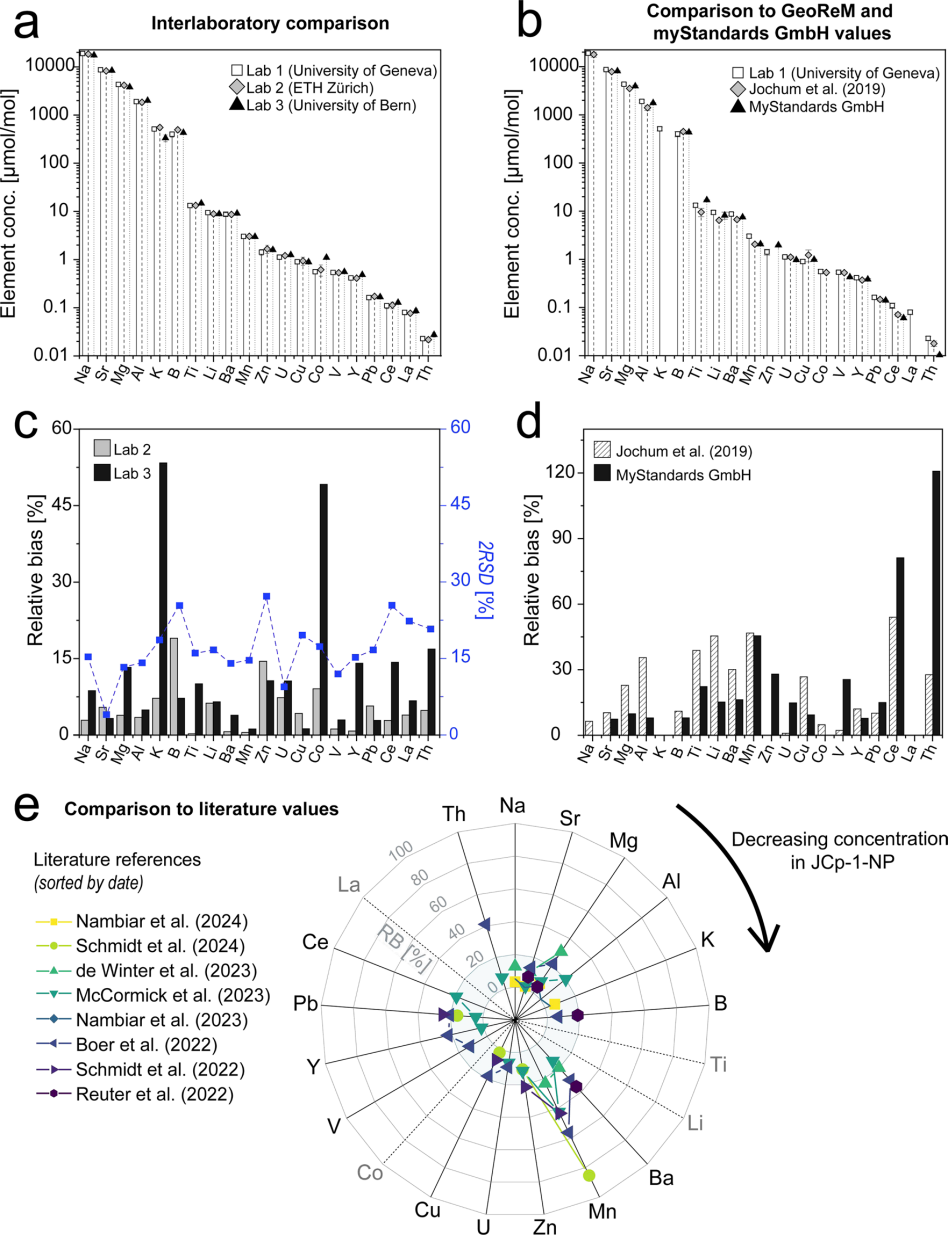
Quality control assessment of the chemical composition of RM JCp-1-NP (myStandards GmbH, batch number: 20170823-60). (**a**) Interlaboratory comparison of chemical composition determined in Laboratory 1, 2 and 3; concentrations are plotted on a logarithmic scale. (**b**) Comparison of Laboratory 1 results with reference values reported by Jochum *et al*.^4^ and values provided by the manufacturer (myStandards GmbH); concentrations are shown on a logarithmic scale. (**c**) Analytical performance of Laboratory 1 results expressed as precision (2RSD) and trueness (relative bias [%]) relative to Laboratory 2 and 3. (**d**) Relative bias of Laboratory 1 results compared to GeoReM and manufacturer reference values. (**e**) Relative bias (RB) assessment (%) of Laboratory 1 values relative to a compilation of published LA-ICP-MS data (provided in Figshare table 6^19^).

We observe no long-term temporal trends in relative bias or precision for any measured E/Ca ratios across the different sessions, indicating that the reference material is stable over time and not prone to changes or alteration while being stored in an air-tight container over silica beads to ensure a dry environment. The precision of combined line and spot analysis in Laboratory 1 was <30% (2RSD) per element (Fig. 2a). The median precision across all elements was around ≈17 ± 6% (2RSD). We can confirm the finding that the precision of elements within the natural RM is dependent on their abundance within the RM^4^, with lower abundance elements showing a higher uncertainty (Fig. 2a).

### Interlaboratory comparison

To compare the results from three participating laboratories, the relative bias (RB) was calculated as the deviation of the mass fractions measured at the collaborating laboratories 2 and 3 from the measured values obtained at Laboratory 1. The result of the interlaboratory comparison showed no systematical deviation between Laboratory 2 and Laboratory 3, with most E/Ca displaying a relative bias of <10% (Fig. 2a). While results from Laboratory 2 exhibited a median deviation of ≈4% ± 5% (RB), the acquired mass fractions from Laboratory 3 deviated by approximately 8% ± 14% (RB) from results obtained from Laboratory 1.

### Comparison to the GeoReM listed values and published values by the RM manufacturer

The comparison of Laboratory 1 data with LA-ICP-MS data by Jochum *et al*.^4^ (GeoReM values), and with published LA-ICP-MS values provided by the manufacturer revealed non-uniform deviations from the reported values (Fig. 2b). On average, E/Ca ratios reported by Jochum *et al*.^4^ differed by ≈23% ± 17% (RB), while Laboratory 1 values deviated from the manufacturer’s by ≈15% ± 30% (RB). High relative deviations from measured to reported values (>40% RB) were observed for Li/Ca, Mn/Ca, and Ce/Ca when compared to the GeoReM values, and for Mn/Ca, Ce/Ca and Th/Ca relative to the manufacturer’s values. However, it is important to note that Jochum *et al*.^4^ used different analytical conditions and did not analyse a nano-powder pellet produced by myStandards GmbH, but used a custom-pressed JCp-1-NP of their own. Contamination during the milling process could have altered the composition of the pellet (e.g.^4,5^) resulting in a composition different to the material of this study.

### Comparison to other published reference values

A compilation of eight publications that conducted LA-ICP-MS analyses on nano-powdered JCp-1 revealed large variability in reported values (Fig. 2c). While our values deviated from published values on average by 12% ± 11% (RB), element-specific deviations between studies spanned up to two orders of magnitude (e.g., the relative bias in Al/Ca ranges from 0.3% to 20%). Most of the measured E/Ca ratios agreed (<20% RB) with previously published data (Fig. 2c). Although nano-powdered pellets show an improved homogeneity^4^ the trace element variability in the relative deviation to the reference of JCp-1-NP caused by chemical heterogeneity within coral skeletons^31^ and with that to the pellet cannot not be fully excluded. Further, counting statistics that are related to the mass fraction of the element of interest in the pellet which leads to lower observed measurement uncertainties for elements that are present in higher mass fraction.

## Data Availability

The dataset is available at Figshare (10.6084/m9.figshare.29682554)^19^.

## Code availability

The R code is available at Figshare (10.6084/m9.figshare.29682554)^19^.
